# Immunomodulatory Properties of Mesenchymal Stromal Cells Can Vary in Genetically Modified Rats

**DOI:** 10.3390/ijms22031181

**Published:** 2021-01-25

**Authors:** Natalie Vallant, Bynvant Sandhu, Karim Hamaoui, Maria Prendecki, Charles Pusey, Vassilios Papalois

**Affiliations:** Department of Transplant Surgery, Faculty of Medicine, Imperial College London, London SW7 2AZ, UK; b.sandhu@imperial.ac.uk (B.S.); karim.hamaoui08@imperial.ac.uk (K.H.); m.prendecki@imperial.ac.uk (M.P.); c.pusey@imperial.ac.uk (C.P.); vassilios.papalois@nhs.net (V.P.)

**Keywords:** mesenchymal stem cells, cytokines, transplantation

## Abstract

Mesenchymal Stromal Cells (MSC) have been shown to exhibit immuno-modulatory and regenerative properties at sites of inflammation. In solid organ transplantation (SOT), administration of MSCs might lead to an alleviation of ischemia-reperfusion injury and a reduction of rejection episodes. Previous reports have suggested ‘MSC-preconditioning’ of macrophages to be partly responsible for the beneficial effects. Whether this results from direct cell-cell interactions (e.g., MSC trans-differentiation at sites of damage), or from paracrine mechanisms, remains unclear. Immunosuppressive capacities of MSCs from donors of different age and from genetically modified donor animals, often used for in-vivo experiments, have so far not been investigated. We conducted an in vitro study to compare paracrine effects of supernatants from MSCs extracted from young and old wild-type *Wystar-Kyoto* rats (*WKY-wt*), as well as young and old *WKY* donor rats positive for the expression of green fluorescent protein *(WKY-GFP)*, on bone marrow derived macrophages (BMDM). Expression levels of *Mannose receptor 1* (*Mrc-1*), *Tumor necrosis factor α* (*TNFα*), *inducible NO synthase* (*iNos*) and *Interleukin-10* (*IL-10*) in BMDMs after treatment with different MSC supernatants were compared by performance of quantitative PCR. We observed different expression patterns of inflammatory markers within BMDMs, depending on age and genotype of origin for MSC supernatants. This must be taken into consideration for preclinical and clinical studies, for which MSCs will be used to treat transplant patients, aiming to mitigate inflammatory and allo-responses.

## 1. Introduction

Mesenchymal Stromal Cells (MSC) are multipotent, self-renewing cells which have been shown to exhibit immuno-modulatory, anti-inflammatory and regenerative capacities at sites of inflammation [1,2,3]. MSCs can be easily isolated from various types of tissues, such as the bone marrow, the umbilical cord or adipose tissue, and can furthermore be differentiated into osteoblasts, adipocytes and chondrocytes in vitro [4]. Therefore, MSCs have been used as an experimental therapeutic agent in graft-versus-host disease (GVHD) [5] and Crohn’s disease [6]. Reported safety properties of MSCs have initiated interest on their administration at or around the time of solid organ transplantation (SOT), with an aim to alleviate ischemia reperfusion injury as well as the risk for subsequent rejection episodes [7,8,9]. Although it was previously assumed that MSCs act by differentiating into functional cells [10], evidence suggests that MSCs are more likely to act in a paracrine manner [11]. Via the secretion of cytokines, growth factors and prostaglandins with immune-modulatory and regenerative function, MSCs may target resident cells. MSCs secrete a range of anti-inflammatory factors, including Interleukin-10 (IL-10), Transforming growth factor-beta (TGF-beta), hepatocyte growth factor (HGF), nitric oxide (NO), Histocompatibility antigen-G (HLA-G) and Prostaglandin E2 (PGE2), that are all indicated to play some role in their immunomodulatory effect [9,12,13]. As MSCs are a mixed population of cells, there may be subtypes of cells with enhanced immunosuppressive capacity [14]. Several reports have suggested ‘MSC-preconditioned’ macrophage activation to be partly responsible for the beneficial effects [15,16,17]. Whether these result from direct cell-cell interactions in the sense of an MSC trans-differentiation at sites of damage, or from paracrine mechanisms, remains unclear.

In SOT, macrophages, amongst other cells, are key initiators of the early innate immune response contributing to ischemia reperfusion injury (IRI), by sending inflammatory signals to other effector cells as well as by a release of soluble mediators. Production of cytokines and chemokines then contributes to an influx of leukocytes, and further promotion of inflammation. Equally however, following activation, macrophages and dendritic cell subpopulations subsequently contribute to the resolution of injury [18]. Depending on the microenvironmental signals, two major phenotypical/functional sub-populations have been identified. The M1 macrophage represents an activated/inflammatory phenotype, whereas the M2 macrophage represents an activated/regenerative phenotype [19]. Therefore, and due to the fact that in vitro macrophage models are very well established, we aimed to investigate the effects of supernatants from different MSC phenotypes on macrophages in vitro.

Several research groups around the world are investigating the effects of MSCs as a therapeutic agent prior to or after SOT, using systemic infusion techniques, or ex-vivo organ machine perfusion techniques in order to precondition organs prior to transplantation. In animal studies, with an aim to trace cells after experiments, often MSCs from genetically modified donors are used, e.g., transgenic animals positive for the expression of green fluorescent protein [20], which for us, raised the question whether any genetic modification, or even a variation in age of donors would have an influence on the immunomodulatory capacities of extracted MSCs. So far, these genetic determinants have not been investigated. We hypothesized, that genetic modification of donor rats for MSCs, as well as the donor age, might have an influence on the immunosuppressive capacities of MSCs. Therefore, our objective was to conduct an in vitro study to investigate paracrine effects of supernatants from MSCs extracted from genetically modified donors and age groups on BMDMs with or without prior inflammatory stimuli.

## 2. Results

**Figure A1:**
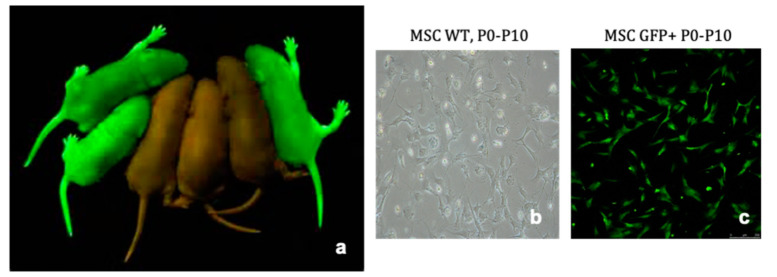
*Wystar Kyoto wildtype (WKY-wt)* and transgenic *WKY rats positive for green fluorescent protein (WKY-GFP)* (**a**) were used as bone marrow donors for the extraction of wildtype MSCs (**b**) and GFP+ MSCs (**c**). Pictures of cells were taken using light microscopy at a 20× magnification for wildtype cells of all donors and for all passages. In (**b**) one representative picture of WKY-wt cells in culture, at a 20× magnification, from a young donor in passage 5 is shown. Using a confocal microscope to detect the green fluorescence in GFP+ cells, in (**c**), a representative picture of WKY-GFP cells in culture, at a 20× magnification, is shown. Cells were in passage 5 and from a young donor rat (6 weeks of age). Strong green fluorescence was present throughout all passages (**c**). Microscopic features were similar for both age groups and throughout passages.

**Figure A2:**
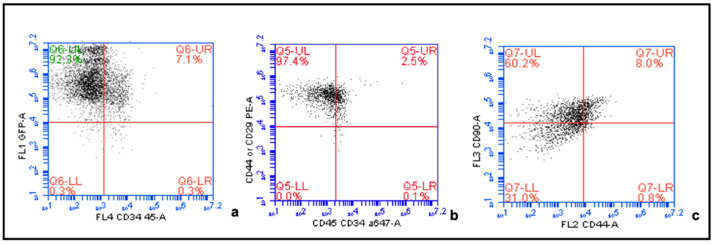
Flow cytometry of extracted MSCs to confirm MSC phenotype. Results of Flow cytometry for MSCs after staining for the surface markers CD29, CD34, CD44, CD45 and CD90. GFP positivity was confirmed by gating for GFP and 92% of those MSCs were negative for CD34 and CD45 (**a**). 97% of all MSCs were positive for CD44 or CD29 whilst being negative for CD45 and CD34 (**b**). 60% of all MSCs were furthermore positive for CD90 and CD44 (**c**).

**Figure A3:**
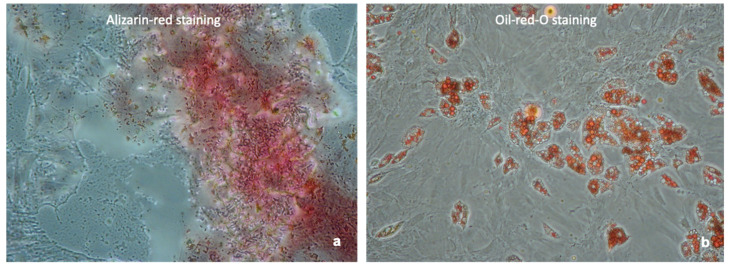
Confirmation of MSC differentiation into osteocytes and adipocytes. 10× magnification of MSCs after stimulation with osteogenic and adipogenic differentiation media. Alizarin-red-staining for cells after osteogenic differentiation is shown in (**a**). Oil-red-O staining for adipocytes after differentiation is shown in (**b**).

MSCs extracted from the bone marrows of *GFP positive rats*, showed green fluorescence, confirmed by confocal microscopy, and by flow cytometry. Green fluorescence was strong and detected in all passages (P1-P10, Appendix A Figure A1 and Figure A2). MSCs from *WKY-wt* and *GFP+ rats* were demonstrated to be plastic adherent, to express the cell surface markers CD44, CD29 and CD90, to lack expression of the hematopoietic markers CD45 and CD34 (Appendix A Figure A2), and to differentiate into adipocytes and osteocytes in culture upon stimulation (Appendix A Figure A3). Results from the MTT viability assay suggested no significant cell death of macrophages after over-night treatments with respective supernatants used for supernatant transfer experiments. The optical densities (ODs) read after performance of the assay were in between 0.88 and 1.09 for all groups, indicating for treated macrophages to be viable. Due to the high amount of data, for each cytokine, only significant differences or non-significant differences with a clear trend for analyzed groups and subgroups will be demonstrated.

### 2.1. Mannose Receptor-1 (Mrc-1)

**Figure 1:**
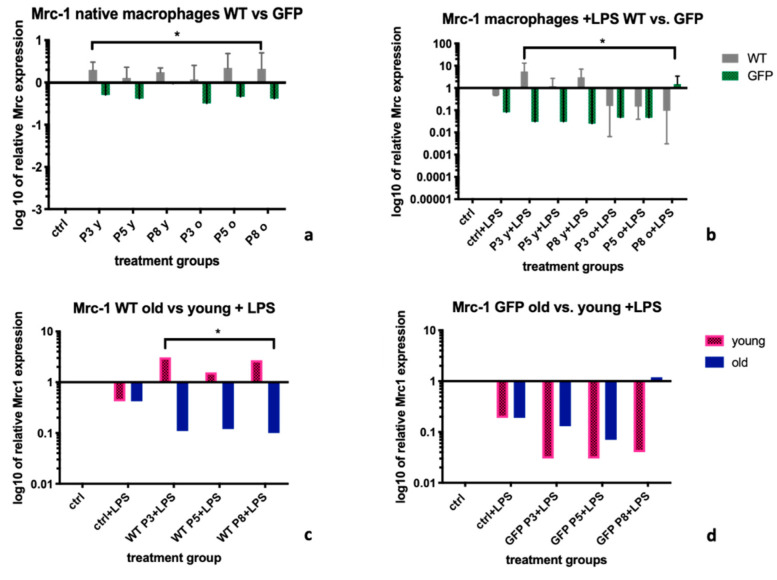
*Mrc-1* expression levels in BMDM after treatment with MSC-wildtype versus MSC-GFP+ supernatants of different age groups and passages. The graph shows the influence of different supernatants from Mesenchymal Stromal Cells on the expression levels of *Mannose receptor-1 (Mrc-1*) in BMDM extracted from a *Wistar Kyoto wildtype (WKY-wt) rat*. Supernatants from MSC-wt led to a significantly stronger upregulation of *Mrc-1* than supernatants from MSC-GFP+ cells, both in native BMDM (**a**), as well as in macrophages after an inflammatory stimulus with Lipopolysaccharide (LPS) (**b**). Furthermore, for supernatants from MSC-wt cells, the difference between the effects from young versus old cell supernatants were significant (**c**), which was not the case within MSC-GFP+ supernatant treatment groups (**d**). Relative mRNA expression levels of *Mrc-1* were calculated using real time (RT)-PCR and the ΔΔCt-method was used for calculations, with untreated BMDM as control and *HPRT-1* as housekeeping gene. Statistical analysis was performed using Mann Whitney U test. Statistical significance: no statistical significance (ns; no asterisk); *p <* 0.05 (*); *B*MDM = Bone marrow derived macrophage, *Mrc-1= Mannose receptor-1*, WT= supernatants coming from MSC-wild-type cells, GFP *=* supernatants from MSC-GFP+ cells. P1-8= passages 1-10. *o* = supernatants from cells from donor rats > 6 months of age, *y* = supernatants from cells from donor rats < 6 months of age.

Supernatants from MSC-wt cells up-regulated *Mannose receptor-1 (Mrc-1)* expression levels within BMDMs more effectively than supernatants from GFP+ MSCs. This was significant for non- stimulated BMDMs (-LPS), with a median *Mrc-1* expression of 1.74 (IQR: 1.18; 2.12) in the MSC-wt group vs. 0.46 in the MSC GFP+ group (IQR: 0.41; 0.94, *p =* 0.03, Figure 1a), as well as for LPS stimulated BMDMs (+LPS), with a median *Mrc-1* expression of 0.73 (IQR: 0.15; 2.85) in former vs. 0.05 (IQR: 0.03; 0.77) in latter group (*p =* 0.02; Figure 1b). Only treatment with MSC-wt supernatants from young donors, not MSC-wt supernatants from old donors nor from any MSC GFP+ supernatants, led to a reversal of the expected down-regulation of *Mrc-1* (Figure 1a–d). When further analyzed, within the LPS treated BMDM group, supernatants from young MSC-wt cells were significantly more potent to upregulate *Mrc-1* than supernatants form old MSC-wt cells. Median expressions levels were 1.56 (IQR: 0.71; 2.9) in the former, vs. 0.12 (IQR: 0.11; 0.71) in the latter group (*p =* 0.04, Figure 1c). There was no difference in expression levels between BMDMs treated with MSC-GFP+ supernatants from young or old donors. Median *Mrc-1* expression levels were 0.04 (IQR: 0.03; 0.59) in the MSC-GFP+ young group vs. 0.19 (IQR: 0.1; 1.09), respectively (*p =* 0.21, Figure 1d).

### 2.2. Inducible Nitric Oxide Synthase (iNOS)

**Figure 2:**
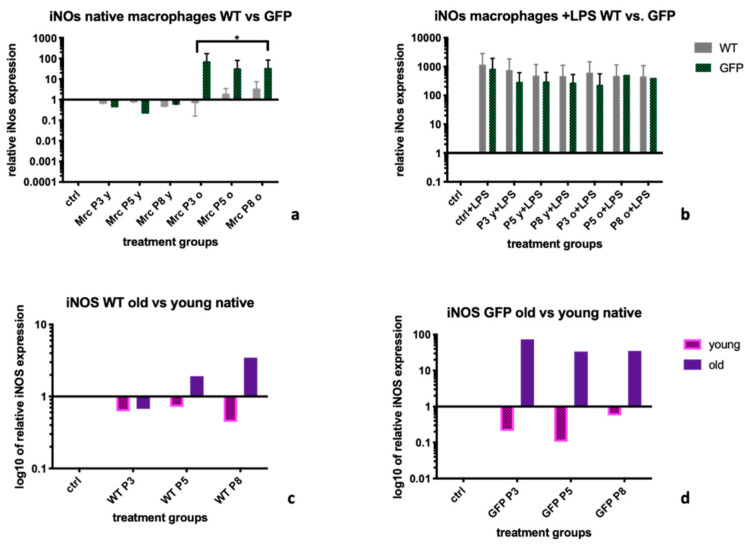
*iNOS* expression levels in BMDM after treatment with MSC-wildtype versus MSC-GFP+ supernatants of different age groups and passages. The graph shows the influence of different MSC supernatants on the expression levels of *Inducible Nitric Oxide Synthase (iNOS)* on BMDM coming from a WKY-wt rat. Supernatants from MSCs extracted from MSC-wt donors led to lower *iNOs* expression levels than supernatants from MSC-GFP+ cells. Furthermore, within supernatants from older donors, this difference was significant (**a**). After LPS stimulation of BMDM, supernatants from GFP+ cells and supernatants from WT cells had similar effects on BMDMs in culture (**b**). Within supernatants from both, MSC-wt and MSC-GFP+ supernatants, young groups inhibited expression of iNOS more efficiently in native BMDM than supernatants from MSCs extracted from old donors, however without statistical significance (**c**,**d**). Statistical evaluation: Mann-Whitney *U* test. mRNA levels were calculated using RT-PCR and the ΔΔCt-method. Untreated macrophages served as control and *HPRT-1* was the housekeeping gene. Statistical significance: no statistical significance (ns; no asterisk); *p <* 0.05 (*). BMDM = bone marrow derived macrophages, *iNOS = Inducible Nitric Oxide Synthase*, WT= supernatants coming from wild-type cells, GFP *=* supernatants from GFP+ cells. Old = supernatants from cells from donor rats >6 months of age, young = supernatants from cells from donor rats < 6 months of age.

Non- stimulated BMDM (-LPS) treated with supernatants from young MSC donors expressed lower amounts of *Inducible Nitric Oxide Synthase (iNOS)* than when treated with supernatants from old donors. Median expression levels were 0.67 (IQR: 0.49; 0.93) for MSC-wt young supernatant treatment vs. 1.46 (IQR: 0.76; 3.1) MSC-wt old supernatant treatment (*p =* 0.25), and 0.39 (IQR: 0.13; 0.89) for MSC-GFP+ young vs. 34.47 (IQR: 9.17; 63.8) MSC-GFP+ old supernatant treatment (*p =* 0.06; Figure 2a). Looking at LPS stimulated BMDMs, *iNOS* expression levels after treatment with supernatants from GFP+ cells were lower (not statistically significant) than after treatment with supernatants from wt-MSCs, in all age groups and passages, with median expression levels of 482.9 (IQR: 457.7; 723.3) in the WT group vs. 301.1 (IQR: 244.2; 495.6), *p =* 0.14 (Figure 2b). Within the untreated BMDM group, we observed differences between supernatants from old MSC donors and young MSC donors within both, the MSC-wt group (1.46 (IQR: 0.76; 3.08) vs. 0.67 (IQR: 0.49; 0.93), *p =* 0.14, Figure 2c), as well as the MSC-GFP+ group (34,47 (IQR: 9.18; 63.8) vs. 0.39 (IQR: 0.13; 0.89), *p =* 0.057, Figure 2d).

### 2.3. Tumor Necrosis Factor α (TNFα)

**Figure 3:**
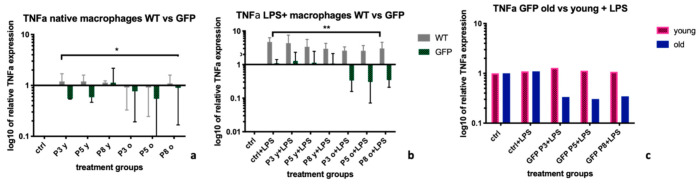
*TNFα* expression levels in BMDMs after treatment with MSC-wildtype versus MSC-GFP+ supernatants of different age groups and passages. The graph shows the influence of different supernatants from Mesenchymal Stromal Cells on the expression levels of *Tumor necrosis factor α (TNFα)* on BMDMs extracted from a *WKY-WT rat*. Supernatants from MSCs extracted from GFP+ donors led to a significantly better reduction of *TNFα* expression levels after stimulation with lipopolysaccharide (LPS) than supernatants from WT-MSCs (*p =* 0.04, calculated using Mann-Whitney U test, (**a**)). Particularly supernatants from older *GFP+ rats* led to a decrease of *TNFα* expression levels (**b**). Within the MSC-GFP+ treated group, *TNFα* expressions were particularly downregulated after treatment with supernatants coming from the older donors, when compared to supernatants from younger donors (**c**). mRNA levels were calculated using RT-PCR and the ΔΔCt-method. Untreated BMDM served as control and *HPRT-1* was the housekeeping gene. Statistical significance: no statistical significance (ns; no asterisk); *p* < 0.05 (*); *p* < 0.005 (**). BMDM= bone marrow derived macrophages, *TNFα* = *Tumor necrosis factor α*, WT = supernatants coming from wild-type cells, GFP *=* supernatants from GFP + cells. Old = supernatants from cells from donor rats > 6 months of age, young= supernatants from cells from donor rats < 6 months of age.

Native BMDMs (-LPS) showed significantly lower relative *TNFα* expression after treatment with supernatants from MSC-GFP + than after treatment with supernatants from MSC-wt, with median expression levels of 0.77 (IQR:0.55; 1) vs. 1.1 (IQR:0.92; 1.2), *p =* 0.019 (Figure 3a). This result was even more significant after LPS stimulation of BMDM, with mean expression levels of 1.04 (IQR: 0.33; 1.23) in former vs. 2.98 (IQR: 2.58; 4.1) in latter group (*p =* 0.002, Figure 3b). Within the MSC-GFP+ treated group, *TNFα* expressions were particularly downregulated after treatment with supernatants coming from the older donors, when compared to supernatants from younger donors, with median expression levels of 0.35 (IQR: 0.32; 1.05) in former vs. 1.1 (IQR: 1.04; 1.28) in latter group (*p =* 0.06), there was however no statistically significant difference between young and old as treatment groups (Figure 3c).

### 2.4. Interleukin-10 (IL-10)

**Figure 4:**
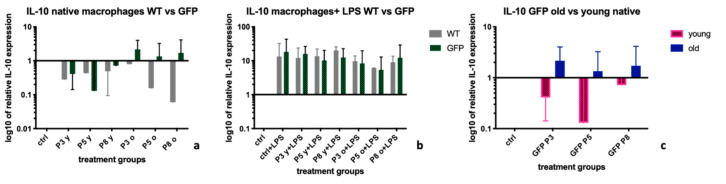
*IL-10* expression levels in BMDMs after treatment with MSC-wildtype versus MSC-GFP+ supernatants of different age groups and passages. Figure 4. the graph shows the influence of different MSC supernatants on the expression levels of *Interleukin-10 (IL-10*) on BMDMs extracted from a *WKY-WT rat*. Supernatants from MSCs from *GFP+ donor rats* led to a higher upregulation of *IL-10* expression levels in native BMDMs than supernatants from WT-MSCs (**a**). Particularly supernatants from older *GFP+ rats* led to an increase of *IL-10* expression levels (**b**). In untreated BMDMs, results indicated a more potent up-regulation of *IL-10* after treatment with supernatants from old MSC donors in comparison to their young equivalents (**c**). mRNA levels were calculated using RT-PCR and the ΔΔCt-method. Untreated macrophages served as control and *HPRT-1* was the housekeeping gene. Statistical significance: no statistical significance (ns; no asterisk); BMDM = bone marrow derived macrophages, *IL-10 = Interleukin-10*, WT = supernatants coming from wild-type cells, GFP *=* supernatants from GFP+ cells. Old = supernatants from cells from donor rats > 6 months of age, young = supernatants from cells from donor rats < 6 months of age.

Treatment of non-stimulated macrophages (-LPS) with supernatants from GFP positive MSCs led to a more potent up-regulation of *IL-10* expression than treatment with supernatants from wt-MSCs (Figure 4a). The mean expression levels of *IL-10* were 0.43 (IQR: 0.15; 0.8) in wt-MSC treated BMDMs vs. 1 (IQR: 0.41; 1.72, *p* = 0.14) in GFP+MSC treated BMDMs. After treatment of BMDMs with LPS, *IL-10* was up-regulated in the control groups (Figure 4b), and there was no difference overall between the two treatment groups (MSC-wt and MSC-GFP+). Median expression levels were 10.9 (IQR: 6.83; 13.52) in the MSC-wt group vs. 11.25 (IQR: 6.13; 14.94), *p =* 0.98 (Figure 4b). In untreated BMDMs, results indicated a more potent up-regulation of *IL-10* after treatment with supernatants from old MSC donors in comparison to their young equivalents. Statistical evaluation showed however no significance of the result, with mean levels of 1.53 (IQR: 1.09; 2.05) after treatment with MSC-GFP+ old supernatants vs. 0.56 (IQR: 0.2; 0.93) after treatment with MSC-GFP+ young supernatants (*p =* 0.057, Figure 4c).

## 3. Discussion

The immunosuppressive properties of MSCs have been extensively studied both in vitro and in animal models of immune-mediated disorders. Clinical trials are currently underway in which MSCs are employed to treat various human immunological diseases as well as inflammatory processes, e.g., ischemia reperfusion injury [21]. The molecular mechanisms leading to immunomodulatory and anti-inflammatory effects of MSCs on different immune cells are still unclear. In that context, ‘MSC- preconditioned’ macrophage activation has been proposed to play a role by some groups in the past [15,16,22]. In order to investigate mechanisms of actions of MSCs, research groups focus on labelling of MSCs prior to their use in in-vivo experiments. Methods of labelling include transfection techniques applied on the cells, or use of transgenic donor animals, classically positive for green fluorescence [20]. Whether genome interfering labelling methods could alter the function, e.g., immunomodulatory capacities of extracted MSCs, has however not been investigated so far. Indeed, the creators of the *GFP+ transgenic WKY rats* used for our experiments, cannot exclude an effect of the genetic manipulation on splicing machineries within the genome, and hence, functional alterations further downstream [20].

A study conducted by Behmoaras et al. in 2014 found, that soluble factors present in supernatants of mesangial cells (MC) from *WKY rats* could differentiate BMDM into active/inflammatory M1, or active/regenerative M2 phenotypes, depending on the genetic background [23] of the MCs used. MCs and MSCs are of similar origin, therefore we hypothesized to potentially find similar differences in MSCs extracted from donors with a genetic modification. Furthermore, we hypothesized, that not only a genetic modification of MSC donors might influence the effect of MSCs on BMDMs in vitro, but also the age of respective donors. Studies have demonstrated that properties and functionalities of MSCs can be influenced by intrinsic factors including aging and that MSCs extracted from young and elder individuals have diverse properties [24,25]. In regards to MSCs as a potential therapeutic agent to alleviate ischemia-reperfusion injury in solid organ transplantation, an aim would be to identify the MSC geno- and phenotype with the most promising anti-inflammatory potential. To our knowledge this is the first study investigating MSC lineage and donor animal age.

Mrc-1 in unstimulated BMDMs was up-regulated after treatment with supernatants from MSC-wt cells, not from MSC-GFP+ cells (Figure 1a). After LPS stimulation, the Mrc-1 downregulation within the control groups was only reversed by supernatants from MSC-wt cells, not from MSC-GFP+ cells (Figure 1b). We furthermore found this effect to be stronger after treatment of LPS stimulated BMDM with supernatants from young MSC-wt cells (Figure 1c). Therefore, in regard to Mrc-1 as marker for the polarization of BMDM, and Mrc-1 upregulation to indicate presence of the more regenerative M2 macrophage phenotype, we found supernatants from young MSC-wt donors to have the most anti-inflammatory effect.

For iNOs, a marker for a more pro-inflammatory BMDM phenotype M1, we observed a better down regulation after treatment with supernatants from MSC-wt than from MSC-GFP+ cells, which was significant for supernatants from old donors (Figure 2a). Sub-group analysis did however not show significant differences between effects from supernatants from young cells versus old cells, for neither of the two, MSC-wt or MSC-GFP+ genotypes of MSCs (Figure 2c,d). Interestingly, after stimulation of BMDM with LPS, none of the supernatant treatments led to an efficient downregulation of iNOS (Figure 2b). We suggest that the capacity for anti-inflammatory effects by chemokines in supernatants might also be influenced by the inflammatory milieu the BMDM are in, and that multiple factors contribute to this. Without cells present in the supernatants, the anti-inflammatory capacity might be too weak to counteract in a pro-inflammatory environment.

TNFα is a key feature of activated/inflammatory M1 macrophages [26,27]. Contrary to findings for iNOS, supernatants from MSC-GFP+ cells, led to significantly better suppression of TNFα expression levels, both in native (Figure 3a) and LPS stimulated macrophages (Figure 3b), than supernatants from MSC-wt cells. The best down-regulation was achieved by supernatants from MSC-GFP+ old cells (Figure 3c). This confirms our theory that supernatants from cells of genetically modified donors can produce different chemokine patterns and therefore, regulate different cytokines in different ways. It might also be dependent on the microenvironment and the activation state of the macrophage, which effect will be observed.

*IL-10*, as a marker for the anti-inflammatory M2 macrophage phenotype [28], was more up-regulated by supernatants from MSC-GFP+ cells in untreated macrophages, than by supernatants from MSC-wt cells (Figure 4a), without reaching statistical significance. Especially supernatants from old MSC-GFP cells seemed to up-regulate *IL-10* expression levels, however in a sub-group analysis between supernatants from young and old MSC-GFP+ cells, no significant difference was detected (Figure 4c). Also after LPS stimulation of macrophages, we did not observe any essential differences in *IL-10* expression levels *(*Figure 4b).

Limitations of our study are that the supernatant transfer experiment was repeated three times, respective PCRs were repeated twice, and each sample was run in duplicates. Therefore, we acknowledge the statistical challenges and the resulting range of results, with sometimes high interquartile ranges. However, we think that the number of repetitions of the experiment as well as of PCR reactions, can also be acknowledged as a strength of the study.

In summary, we have observed similar results as described by Behmoaras et al. for mesangial cells. In our case, different paracrine effects were observed for supernatants from MSCs from a genetically modified donor rat, expressing GFP under the control of elongation factor 1α, as well as from MSCs of rats of different age groups. Different passages showed similar results. Looking at *Mrc-1* and *iNOS* regulations to start with, it seemed like MSC-wt cells were more promising as inhibitor cells of inflammatory cascades in a paracrine manner. However looking at *TNFα* and *IL-10* expression levels, supernatants from MSC-GFP+ cells seemed to be more promising in regards to promotion of anti-inflammatory cascades within macrophages.

For future experiments, it would be sensible to measure the exact concentrations of cytokines present in the supernatants, and also, to perform a similar experiment on a direct cell-cell interaction level in parallel. This might help to further understand the mechanisms of action of MSCs. Nevertheless, different phenotypes of MSCs depending on their age and the presence of genetic modifications of donor animals should be considered in in vivo studies.

## 4. Materials and Methods

All studies were performed with Ethical approval and approval under the Animal Scientific Procedures Act (1986). Experiments were performed under Project Licence Number PB1C4696D, granted by the Home Office, UK.

### 4.1. Extraction and Culture of Bone Marrow Derived Mesenchymal Stromal Cells

MSCs were extracted from the bone marrow of *wild-type Wystar Kyoto rats (WKY-wt)* and from *Wystar Kyoto rats* positive for the expression of green fluorescent protein *(WKY-GFP+)*. This specific rat had been genetically modified to express GFP under the control of elongation factor 1α, by Dr. Anna Garcia-Diaz et al [20]. Donor rats were either 6 weeks (young), or 6 months (old) of age, respectively. After sacrifice of the donor rats according to the Schedule 1 protocol, femurs and tibias were retrieved and bone marrow was flushed into fresh Falcon tubes using sterile Hank’s Balanced Salt Solution (HBSS; Gibco, Thermo Fisher Scientific, Waltham, MA, USA) at 4 °C. Cell suspensions were washed twice with HBSS, supernatants were discarded and pellets containing cells were resuspended in 1 mL of MesenCult^TM^ MSC Basal Medium (StemCell Technologies, Cambridge, UK) before being added into flasks containing 24 mL full MSC culture medium containing MSC stimulatory supplement and 0.5% Penicillin/Streptomycin (Invitrogen, Carlsbad, CA, USA). At a confluence of 80%, cells were trypsinized, counted and split.

### 4.2. Confirmation of MSC Phenotype

MSC phenotype for all MSC cultures was proven by demonstration of their differentiation capacities into adipocytes and osteocytes. Rat MSC Adipogenic Bullet kit medium (Lonza, Basel, Switzerland) was added to the cells for adipogenic induction, and rat Osteogenic Bullet kit medium (Lonza) for osteogenic differentiation. Differentiation of the cells into adipocytes was confirmed by Oil-Red-O staining and formation of bone matrix was confirmed by Alizarin Red staining. Furthermore, MSC phenotype was confirmed by Flow cytometry. Cells were shown to express the cell surface markers CD29 (antibody: PE anti-mouse/rat CD29, clone: HMβ1-1, Bio Legend Cat: 102207, Conc: 0.2 mg/mL), CD90 (antibody: PerCP anti-rat CD90/mouse, CD90.1 (Thy-1.1) clone: OX-7, BioLegend Cat: 202512; Lot: B171081, Conc: 0.2 mg/mL), and CD44 (antibody: RPE mouse anti-rat CD44, clone OX-50, Bio Rad Cat: MCA643PE), and to lack the expression of CD34 (antibody: Alexa Fluor 647 anti-mouse CD34, clone: ICO115, Novus Biologicals Cat: NBP2-33076AF647; Conc: 0.75 mg/mL) and CD45 (antibody: Alexa Fluor 647 anti-rat CD45, clone: OX-1, BioLegend Cat: 202212; Conc: 0.5 mg/mL) for MSC phenotyping (Figure A2).

### 4.3. MSC Supernatants

For the purpose of supernatant transfer experiments, for each group and passage of MSC cultures (P1-P10), triplicates of 2.5 × 10^5^ cells were placed into a 24 well plate in 500 µL of MesenCult^TM^ full culture medium (StemCell, Catalogue number Catalog #05513) for 24 h. Thereafter, supernatants from all the wells were collected and stored on −80 °C until used for the experiments. The following groups of supernatants were obtained for experiments:

- **MSC-wt young, P1-P10**: supernatants from MSCs in culture, extracted from a 6 week old WKY-wt rat, passages 1–10.
- **MSC-GFP+ young, P1-P10**: supernatants from MSCs in culture, extracted from a 6 week old WKY-GFP+ rat, passages 1–10.
- **MSC-wt old, P1-P10**: supernatants from MSCs in culture, extracted from a 6 month old WKY-wt rat, passages 1–10.
- **MSC-GFP+ old, P1-P10**: supernatants from MSCs in culture, extracted from a 6 month old WKY-GFP+ rat, passages 1–10.

### 4.4. Extraction of Bone Marrow Derived Macrophages

L929 conditioned culture medium for macrophage culture was prepared and filtered with a sterile filter. Bone marrow derived macrophages were obtained from a *WKY-*wt rat. The animal was sacrificed according to the Schedule 1 protocol. Femurs and the tibias were retrieved, and bone marrow was flushed into a Falcon tube in a laminar flow hood. The content was spun for 5 min at 1500 rpm and a temperature of 4 °C. The supernatant was discarded and the pellet containing the cells was resuspended in 10 mL of HBSS. Red blood cell lysis was performed. Cells were spun again for 5 min at 1500 rpm, 4 °C, the supernatant was discarded and cells were resuspended in 1 mL of full culture media, which per 375 mL contained: 240 mL DMEM (+L-glu), 3 g of Hepes 25 mM, 125 mL L929 conditioned media (25%) and 10 mL Pen/Strep (100 U/mL, 100 ug/mL). The cells were dispensed equally into 3 large petri dishes containing 25 mL BMDM full culture medium and cultured for 5 days in the 37 °C, 5%CO2 incubator. After 5 days, BMDM full culture medium was removed and dissociation buffer was added to cover the surface of the cells before incubating for 10–20 min at 37 °C, 5%CO2. Cells were then collected and spun down for 5 min at 1500 rpm. The supernatants were discarded and 1 mL of BMDM full culture medium was added, respectively, to resuspend the cells. Cells were counted with the use of a haemocytometer and 2.5 × 10^5^ cells per well were seeded into 24 well plates in 400 µL of BMDM full culture medium, respectively. Cells were incubated over night to adhere to the plastic again.

### 4.5. Supernatant Transfer Experiments

After confirmation of the viability of BMDMs in all wells of the 24 well plates by light microscopy, wells were washed with HBSS. Macrophage control samples (Mac ctrl) were treated with 400 µL of the DMEM based medium alone. MSC control samples (Mac MSC ctrl) contained 150 µL DMEM based medium + 250 µL plain MSC growth medium which was freshly prepared in order to rule out that any effect could come from any of the ingredients of the MSC growth medium (MesenCult proliferation kit, Stem Cell Technologies, UK). Cells in treated wells within columns 4–6 of a 24 well plate, underwent treatment with LPS for 1 h, respectively. Wells in columns 1–3 underwent change of media only. Macrophages were treated with 250 µL of the supernatants from MSCs P1-P10. Each well contained 150 µL DMEM based medium and 250 µL of MSC supernatant. Figure A3 shows a scheme of the experiment, highlighting respective treatment groups within the 24 well plates. The plates were left in an incubator at 37 °C and 5%CO_2_ over-night. Thereafter the macrophages were washed with HBSS and the cells from each well were lysed using 250 µL of Trizol reagent for RNA extraction.

### 4.6. qRT-PCR

For each well, RNA extraction was performed with the TRIzol method (Thermo Fisher Scientific, UK). DNA was digested using DNase I (Sigma-Aldrich, UK). The concentration and purity of RNA samples was assessed using the A260:A280 ratio and A260:A230 ratio, respectively using a Nano drop 2000c Spectrophotometer (Thermo Fisher Scientific). Quantitative real time PCR (qRT-PCR) was performed using the iScript Sybr Green Supermix (BioRad, Hertfordshire, UK). Optimal Primer combinations and PCR programmes were tested before performance of the actual qRT-PCR. PCRs were performed in duplicates in 96-well PCR plates (Thermo Fisher Scientific, UK) on an Eppendorf Mastercycler^®^ RealPlex. PCRs were performed with the following protocol:

3 min at 95 °C (hot start), followed by 40 cycles of the following:

15 s at 95 °C denaturation step

20 s at 60.4 °C primer annealing step

40 s at 72 °C elongation step, followed by

Melting curve

Hold at 4 °C

The housekeeping gene *Hypoxanthine-Guanine Phosphoribosyltransferase (HPRT)* was used as positive control. Primer pair sequences for these genes as well as their product lengths are shown in Table 1. All primer pairs were designed using Primer blast (NCBI) and were synthesised by Sigma-Aldrich. The relative quantification of a target gene in the PCR reaction compared to the control was calculated using the comparative Ct method ΔΔCt. Specific primer combinations for qRT-PCR are shown in Table 1.

### 4.7. MTT Viability Assay

The MTT viability Assay was performed on macrophages, mimicking circumstances of respective supernatant transfer experiments, in order to investigate whether the treatment led to cell death that could cause altered results. The MTT (3-(4,5-dimethylthiazol-2-yl)-2,5-diphenyltetrazolium bromide) reagent was diluted 1:10 in culture media. In a 96-well plate, rat bone marrow derived macrophages (BMDM) underwent the same respective treatments as macrophages in 24-well plates for previously described experiments. Supernatant were removed from cells without disturbing the cell sheet and 100μL MTT solution per well were added. Cells were incubated overnight at 37 °C in the incubator. Subsequently an equal volume of SDS MTT solution (10% SDS, 0.01M HCl) was added and incubated at 37 °C for at least 3 h or overnight. The plate was read on an ELISA plate reader at 592 nm and ODs were compared.

### 4.8. Statistical Evaluation

**Figure A4:**
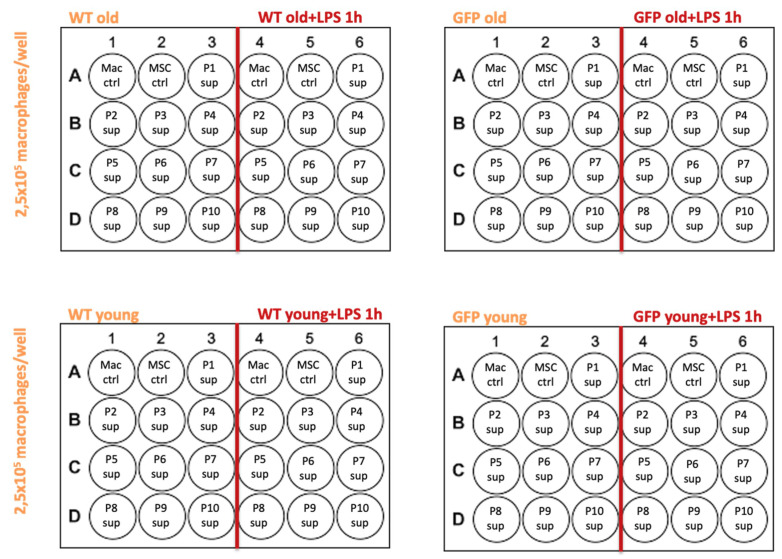
Scheme of in vitro supernatant transfer experiment. The experimental scheme of different in vitro treatments of BMDMs with different supernatants coming from MSCs is illustrated in the Figure. *WT old = Wildtype Wistar Kyoto rat* >6 months of age as a Mesenchymal Stromal Cell (MSC) donor. *WT young = Wildtype Wistar Kyoto rat* <6 months of age as MSC donor. LPS = lipopolysaccharide treatment. *GFP old = transgenic Wistar Kyoto rat positive for ubiquitous expression of green fluorescent protein (GFP)* >6 months of age as MSC donor. *GFP young= transgenic Wistar Kyoto rat positive for ubiquitous expression of green fluorescent protein (GFP)* <6 months of age as MSC donor. Mac ctrl= untreated macrophages in DMEM based medium as control. MSC ctrl = macrophage in DMEM based medium, treated with blank MSC medium. P1-P10 = passage 1–10; sup *=* supernatant.

The supernatant transfer experiment was repeated three times and qRT-PCRs were performed in duplicates and repeated twice, respectively. Relative mRNA expression levels of selected cytokines were calculated using the ΔΔCt method and using the ubiquitously expressed *Hypoxanthine-Guanine Phosphoribosyltransferase (HPRT)* as a housekeeping gene. Relative expressions were calculated in comparison to the respective control samples (e.g., macrophage control, LPS control, macrophage MSC control- see Figure A4). Mean values from the three experiments were calculated, respectively, and statistical analyses between groups were performed using Mann Whitney U test as well as ANOVA. In order to simplify the results, for subgroup analyses, passages were plotted together to result in one mean value, respectively (this resulted in columns without markers for a standard deviation as only one mean value was used for analysis). Statistical analyses were conducted for all subgroups but in this manuscript, with consciousness to an otherwise overwhelming set of data, only the most interesting/significant findings are demonstrated and visualized by graphs. GraphPad Prism was used to perform all statistical analyses.

## 5. Conclusions

Supernatants from MSCs do influence the immunogenic profile of macrophages suggesting chemotactic immunomodulatory effects of the cells. However, results differ between supernatants from MSCs, depending on the presence of genetic modifications of respective donor animals, as well as their age, and the inflammatory milieu they are in. This should be taken into consideration for future preclinical and clinical studies involving MSCs, with an aim to find the most potent cell type tailored to its potential purpose, before using them as an immunomodulatory agent in different situations. In order to find the most promising anti-inflammatory MSC phenotype, further studies are needed.

## Figures and Tables

**Table 1:** Rat specific primer combinations for quantitative real time PCR.

Gene	Sequence	Tm	Product Length (bp)	Concentration Used in PCR
**HPRT for**	GCACGAGGGACTTACCTCAC	63.7 °C	128 bp	100 nM
**HPRT rev**	TAATCACGACGCTGGGACTG	66.2 °C	128 bp	100 nM
**IL-10 for**	TAAAAGCAAGGCAGTGGAGC	64.3 °C	147 bp	100 nM
**IL-10 rev**	TGCCGGGTGGTTCAATTTTTC	69.7 °C	147 bp	100 nM
**TNFα for**	ATGGGCTCCCTCTCATCAGT	65 °C	106 bp	100 nM
**TNFα rev**	GCTTGGTGGTTTGCTACGAC	64.5 °C	106 bp	100 nM
**NOs-2 for**	TCAGGCTTGGGTCTTGTTAGC	65.4 °C	110 bp	100 nM
**NOs-2 rev**	GAAGAGAAACTTCCAGGGGCA	66.5 °C	110 bp	100 nM
**Mrc-1 for**	TGATTCCGGTCGCTGTTCAA	68.9 °C	99 bp	100 nM
**Mrc-1 rev**	GAACGGAGATGGCGCTTAGA	66.5 °C	99 bp	100 nM

## Data Availability

The data that support the findings of this study are available from the corresponding author upon reasonable request.

## Abbreviations

BMDM	Bone Marrow Derived Macrophages
GFP	Green fluorescent protein
GVHD	Graft versus host disease
IL-10	Interleukin-10
ISCT	International Society for Stem Cell Therapy
iNOS	Inducible Nitric Oxide Synthase
MSC	Mesenchymal Stromal Cells
Mrc-1	Mannose receptor-1
TNFα	Tumor Necrosis Factor α
WKY	Wistar Kyoto
WT	Wild type
qPCR	Quantitative polymerase chain reaction
